# Very old patients with cancer admitted to the ICU: outcome and predictive factors of mortality

**DOI:** 10.1186/cc13248

**Published:** 2014-03-17

**Authors:** L Hajjar, M Franca, J Almeida, J Fukushima, F Bergamin, C Zambolim, P Camargo, C Park, E Osawa, M Sundin, R Nakamura, F Galas

**Affiliations:** 1 Instituto do Cancer do Estado de São Paulo, Brazil; 2 Heart Institute, São Paulo, Brazil

## Introduction

The rate of very old patients (≥80 years old) admitted to intensive care has increased in the last years. Older age is associated with a higher prevalence of chronic diseases, including cancer [1]. The aim of the present study was to describe characteristics, outcomes and predictive factors of mortality in very old patients admitted to the ICU.

## Methods

We performed a retrospective analysis of all cancer patients who were 80 years or older admitted to the ICU between January 2009 and December 2012 in a tertiary reference cancer center. Data were collected from medical records.

## Results

A total of 597 patients with cancer were included in the analysis. Hospital mortality was 28.5%. These patients were more likely to have a solid tumor, a localized disease and underwent surgery, chemotherapy or radiotherapy recently. Variables associated with hospital mortality in these patients were: lung cancer, metastatic cancer and at ICU admission vasopressor requirements, acute respiratory failure, low hemoglobin levels, elevated creatinine levels, elevated bilirubin levels, acidosis and hyperlactatemia. By the multivariate analysis, the following factors were independent factors associated with hospital mortality: lung cancer (odds ratio (OR) = 6.3, 95% CI = 2.6 to 15.0, *P *< 0.001), lactate levels on ICU admission (OR = 1.03, 95% CI = 1.02 to 1.04), *P *< 0.001), bilirubin levels on ICU admission (OR = 1.16, 95% CI = 1.4 to 1.30, *P *= 0.007) and creatinine levels on ICU admission (OR = 1.58, 95% CI = 1.35 to 1.85, *P *< 0.001).

## Conclusion

Very old patients with cancer present acceptable rates of survival after ICU admission. Similarly to younger patients, organ function evaluation in the first hours of ICU can predict outcomes in this specific population.
